# Dietary compounds have potential in controlling atherosclerosis by modulating macrophage cholesterol metabolism and inflammation via miRNA

**DOI:** 10.1038/s41538-018-0022-8

**Published:** 2018-07-25

**Authors:** Dongyan Shao, Ziyang Lian, Yichao Di, Lei Zhang, Muhammad shahid riaz Rajoka, Yudan Zhang, Jie Kong, Chunmei Jiang, Junling Shi

**Affiliations:** 1 Key Laboratory for Space Bioscience and Biotechnology, School of Life Sciences, Northwestern Polytechnical University, 127 Youyi West Road, Xi’an, 710072 Shaanxi China; 2 Department of Microbiology and Pathogeny Biology, Xi’an Medical University, 1 Xinwang Road, Xi’an, 710072 Shaanxi China; 3 MOE Key Laboratory of Space Applied Physics and Chemistry, Shaanxi Key Laboratory of Macromolecular Science and Technology, School of Science, Northwestern Polytechnical University, Xi’an, 710072 Shaanxi China

**Keywords:** Nutrition, Cardiovascular diseases

## Abstract

Atherosclerosis (AS) is a typical example of a widespread fatal cardiovascular disease. Accumulation of cholesterol-laden macrophages in the artery wall forms the starting point of AS. Increased influx of oxidized low-density lipoprotein to macrophages and decreased efflux of free cholesterol out of macrophages constitute major factors promoting the development of AS. Inflammation further aggravates the development of AS along or via interaction with the cholesterol metabolism. Many microRNAs (miRNAs) are related to the regulation of macrophage in AS in aspects of cholesterol metabolism and inflammation signaling. Dietary compounds perform AS inhibitory effects via miRNAs in the cholesterol metabolism (miR-19b, miR-378, miR-10b, miR-33a, and miR-33b) and two miRNAs in the inflammation signaling (miR-155 and miR-146a). The targeted miRNAs in the cholesterol metabolism vary greatly among different food compounds; however, in inflammation signaling, most food compounds target miR-155. Many receptors are involved in macrophages via miRNAs, including ABCA1 and ABCG1 as major receptors in the cholesterol metabolism, while nuclear factor-κB (NF-κB) and Nrf2 signaling and PI3K/AKT signaling pathways are targeted during inflammation. This article reviews current literature to investigate possible AS therapy with dietary compounds via targeting miRNAs. Currently existing problems were also discussed to guide further studies.

## Introduction

Atherosclerosis (AS) is one of the major factors contributing to cardiovascular diseases (CVD), which is the leading disease that causes morbidity and mortality all over the world, accounting for approximately 27% of all deaths among males and 32% among females.^1,2^ Macrophage-related cholesterol metabolism and inflammation are two major factors related to the development of AS at different periods, including initiation, progression, and aggression.^3,4^

**Fig. 1:**
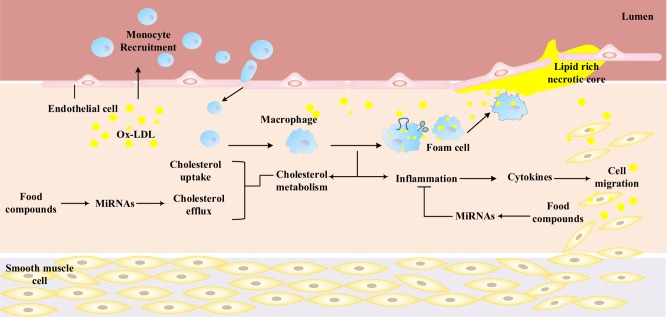
Roles of monocytes/macrophages in promoting AS and the influence of dietary compounds on macrophages. At the initiation of AS, the damage of the endothelium, which is to a certain extent caused by the accumulation of modified cholesterol (ox-LDL), initiates an immune response recruiting monocytes into the subendothelial space, where these cells differentiate into macrophages. The formed macrophages tend to uptake the ox-LDL unlimitedly and become cholesterol-loaded foam cells. These foam cells accumulate to create fatty streaks, which form the central feature of the early stage during the development of atherosclerotic lesions. Further development of macrophage foam cell formation is promoted by escalating inflammation of macrophages and other immunocytes. Overall, macrophage-related cholesterol metabolism and inflammation are two major factors related to the development of AS. Some food compounds could attenuate AS by increasing cholesterol efflux and inhibiting inflammation via targeting miRNAs. AS, atherosclerosis; ox-LDL, oxidized low-density lipoprotein

With regard to the cholesterol metabolism, the trap of modified low-density lipoproteins (mainly oxidized low density lipoproteins (ox-LDL), which are rich in cholesterol) on the vessel wall are a prologue to the formation of AS.^5^ Such an accumulation of ox-LDL causes endothelial damage, which subsequently induces an immunological response that recruits monocytes to the subendothelial space, where these differentiate into macrophages. The formed macrophages tend to uptake the accumulated ox-LDL and become cholesterol-loaded foam cells. These foam cells accumulate to create fatty streaks, which form the central feature of the early stage during the development of atherosclerotic lesions^6^ (Fig. 1). The increased amount of ox-LDL is a result of the disturbed cholesterol metabolism between influx and efflux pathways, which is determined by the expression and activity of corresponding receptors and signal molecules. Any methods to inhibit the trapping of cholesterol in or the promotion of cholesterol efflux from macrophages would have the potential to inhibit the occurrence and the progression of AS.

With regard to AS-related inflammation, the formed foam cells tend to secrete a wide array of cytokines, including proinflammatory mediators, such as chemokines, cytokines, reactive oxygen and nitrogen species, and matrix-degrading proteases.^3^ These proinflammatory mediators can recruit additional monocytes, smooth muscle cells, and other immunocytes such as T cells and dendritic cells to the space around the formed AS lesion.^7^ This leads to further development of atherosclerotic lesions, more amplified inflammation, and finally vascular remodeling and increasing plaque susceptibility to thrombus formation.^4^ Any methods and factors that can inhibit the formation of foam cells or the secretion of proinflammatory mediators may have promising potential for inhibiting the progression and regression of AS.

In recent years, with the discovery of microRNA (miRNA) functions, many miRNAs have been found to control macrophage functions in cholesterol homeostasis and inflammation, therefore regulating the progression of AS.^8–11^ miRNAs are endogenous, short (between 19 and 25 nucleotides long), and highly conserved noncoding RNAs that can principally regulate gene expression at the posttranscriptional level.^12^ It has been reported that miRNAs can regulate several metabolic pathways, including insulin secretion and carbohydrate and lipid metabolism.^13^ miRNAs might bind to as many as 200 gene targets including transcription factors, secreted and transmembrane factors (such as factors known to mediate axon guidance decisions during the neural connectivity), receptors, and transporters.^14,15^ Some miRNAs control endothelial cell (EC), vascular smooth muscle cells, and macrophage functions, thus regulating the progression of atherosclerosis. Recently, many miRNAs have emerged as new regulators of AS. The miRNAs have been found to play important roles in aspects of regulating the macrophage cholesterol metabolism and inflammation and these are summarized in Table 1.

**Table 1:** Currently reported miRNAs involved in the macrophage cholesterol metabolism and inflammation in the progression of atherosclerosis

Function	miRNAs	Targets		Reference
	miR-125a-5p	LOX-1, CD68		37
Cholesterol metabolism	miR-155	LOX-1, CD36, CD68		38
	miR-467b	ACAT1		39
	miR-9	ACAT1		40
	miR-134	ANGPTL4		41
	miR-467b	LPL		42
	miR-33	ABCA1		45, 46
	miR-19b	ABCA1		8
	miR-144, -26, -27a, -758, -148a	ABCA1		52-55
	miR-10b	ABCA1, ABCG1		56, 57
	miR-378	ABCG1		58
Inflammation	miR-146a	IRAK1, TRAF6		36, 88
	miR-155	SMAD2, FOXO3a, SOCS1, SHIP1, BCL6		19, 87, 11
	miR-125a-5p	Unknown		37
	miR-467b	Unknown		42

Many dietary products or dietary supplements have been recently reported to possess potential for development as therapeutic agents,^16^ such as red wine,^17^ propolis,^18^ apigenin,^19^ and berries^20^ that have all shown a certain capability to attenuate the progression of AS. More importantly, some natural micronutrients and nonnutrient components in these foods, such as polyphenols, have been reported to modify the expression of miRNAs related to cholesterol metabolism^21,22^ and/or inflammation^23^ (Fig. 1). Hence, targeting miRNAs using natural dietary components may be a novel, safe, and promising strategy in the prevention and therapy of AS.

**Fig. 2:**
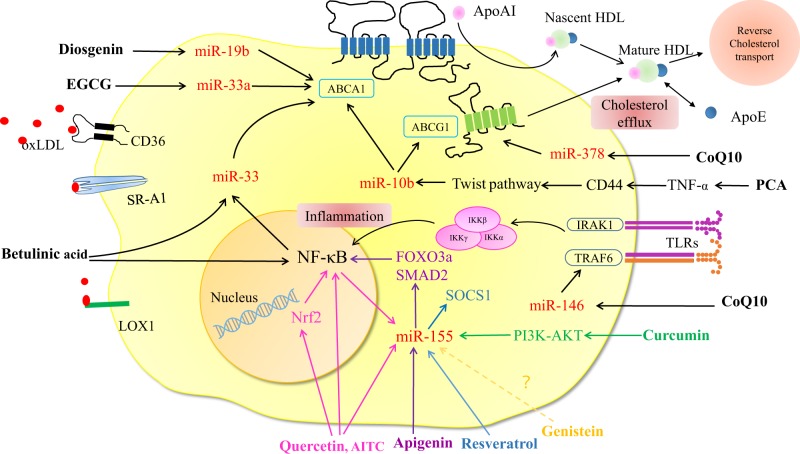
Dietary compounds regulate AS-related macrophage cholesterol metabolism and inflammation via miRNAs. Different food compounds play atheroprotective roles by various miRNAs via different signaling pathways. (1) Diosgenin, EGCG, betulinic acid, CoQ10, and PCA could increase cholesterol efflux by inhibiting miR-19b, miR-33, miR-378, miR-33, and miR-10b, respectively. (2) Quercetin, AITC, apigenin, curcumin, and resveratrol could inhibit inflammatory response, through targeting to miR-155. CoQ10 could regulate inflammation via inhibiting miR-146a. With regard to the macrophage inflammation, different color lines represent the different dietary compounds regulate diverse signaling pathways via different miRNAs. AITC, allyl-isothiocyanate; CoQ10, coenzyme Q10; EGCG, epigallocatechin-3-gallate; PCA protocatechuic acid

This review provides a summary of the current reports related to the correlation among dietary compounds, miRNA expression, and modulation of macrophage lipid metabolism and inflammation in AS, and presents clues for the modulation of AS by dietary compounds via miRNAs. The overall inhibitory effect of dietary compounds on AS via miRNAs is summarized in Fig. 2.

## Dietary compounds inhibit AS by influencing the cholesterol metabolism of macrophage via miRNA

### Cholesterol metabolism of macrophage

It has been widely accepted that excess ox-LDL forms the basic cause of AS because accumulated ox-LDL is trapped in macrophages and then induces the formation of foam cells that tend to accumulate and form initial AS lesions on the vessel wall.^5^ Such cytoplasmic accumulation of ox-LDL in foam cells exacerbates the trap of ox-LDL in blood vessels and promotes the development of AS.^24^ Therefore, the development of AS can be slowed down or controlled in two ways: one is decreasing the uptake of ox-LDL via macrophages; the other one is enhancing the efflux of ox-LDL out of macrophages.

The internalization of ox-LDL by macrophages is mainly mediated by scavenger receptors (SRs). These SRs can be divided into four major classes: SR-A (SR-AI, SR-AII, and SR-AIII), SR-B (CLA-1/SR-BI, SR-BII, and CD36), SR-D (CD68 and SR-PSOX, also known as CXCL16), and the lectin-like oxidized LDL receptor-1 (LOX-1).^2^ Among them, SR-A and CD36 account for 75–90% of the total uptake of ox-LDL.^25^ Intracellular cholesterol in macrophages has no feedback inhibition on the uptake of ox-LDL and thus causes the formation of macrophage foam cells and traps cholesterol in blood vessels.^5^ In contrast, the identification and uptake of native LDL can be negatively regulated by the intercellular cholesterol level via the LDL receptor (LDLR). Expression of the LDLR, and the 3-hydroxy-3-methyl glutaryl-CoA reductase (HMGCR), which dominates the endogenous cholesterol synthesis, are triggered by the sterol regulatory element-binding proteins (SREBP), SREBP-2, and SREBP-1a.^5,26^ Inhibition of SREBP can repress the trap of LDL in macrophages and the accompanying formation of foam cells via decreases of LDLR and HMGCR.

A characteristic feature of foam cells is the cytoplasmic accumulation of cholesteryl ester-rich droplets. Ox-LDL taken up by macrophages is delivered to lysosomes, where its cholesterol ester content is hydrolyzed into free cholesterol by lysosomal acid lipase. Free cholesterol is then transferred to the plasma membrane and be effluxed from the cell or to the endoplasmic reticulum (ER), where it undergoes reesterification with acyl coenzyme A: acyl cholesterol transferase (SOAT; formerly known as ACAT) to cholesteryl fatty acid esters that provide the “foam” of the foam cells. Therefore, efforts aimed at the decrease of ACAT function can be also used as a target for AS control via reducing the formation of cholesterol ester.^27^

As intracellular cholesterol levels increase, endogenous cholesterol biosynthesis, and LDLR expression are repressed through inhibition of the SREBP pathway. However, this mechanism is insufficient to maintain cholesterol homeostasis when continued ox-LDL uptake by SR.^28^ In this case, excessive cholesterol needs to be excreted from cells and transported by high-density lipoprotein (HDL) to the liver for reuse or secretion out of the body. The whole process is called reverse cholesterol transport (RCT)^29^ and enhancing the RCT process has been reported to reduce the occurrence of AS.^30,31^ The efflux of free cholesterol from foam cells is regulated by the ATP-binding cassette transporters A1 (ABCA1) and G1 (ABCG1),^32^ which are induced by the sterol regulated transcription factors, LXR. ABCA1 is a key plasma membrane protein responsible for the transfer of free cholesterol to extracellular acceptor, particularly apoA-I, and then forming HDL. Free cholesterol can also be directly transferred out of cells to HDL through ABCG1. The ABCA1-dependent cholesterol efflux is a critical event in the prevention of excessive cholesterol accumulation in macrophages of the arterial wall and their transformation into foam cells.^33^ Macrophage knockout of ABCA1 in mice has been reported to result in increased free and esterified cholesterol in macrophages and enhanced inflammatory responses.^34^ However, over expression of ABCA1 in macrophages in mice inhibited atherosclerotic lesion progression and the occurrence of atherosclerosis with minimal effects on plasma HDL.^35^ In addition, apoE, a component of plasma lipoproteins, which is secreted by macrophages, aids the promotion of cholesterol efflux and clearance and has anti-inflammatory properties against AS.^36^

### miRNAs involved in cholesterol metabolism of macrophage

With the continuous discovery of miRNAs functions, numerous miRNAs have been identified to be related to the occurrence of AS. MiR-125a-5p has been found to decrease cholesterol uptake in ox-LDL-stimulated monocyte/macrophages via inhibiting the expressions of LOX-1 and CD68.^37^ In contrast, miR-155 promotes the cholesterol uptake in THP-1 macrophages via enhancing the expression of the SRs, LOX-1, CD36, and CD68.^38^ Therefore, miR-125a-5p and miR-155 are potential targets for AS therapy in the trap of ox-LDL process.

For the reesterification of free cholesterol by ACAT in the ER, miR-467b can reduce ACAT1 expression via targeting its 3'UTR in ox-LDL-treatment RAW264.7 macrophages, which affects the CE formation and consequently decreases the AS process.^39^ Similarly, miR-9 could also target human ACAT1 mRNA and functionally decrease foam cell formation of THP-1-derived macrophages.^40^

Lipoprotein lipase (LPL) is a rate-limiting enzyme in catalyzing and hydrolyzing triglyceride, it can promote lipid accumulation and inflammation. Angiopoietin-like 4 (Angptl4), a secreted protein, is an important regulator to irreversibly inhibit LPL activity. It was found that miR-134 could suppress the expression of ANGPTL4 so that activate LPL-mediated lipid accumulation. Furthermore, the levels of proinflammatory cytokine TNF-α, MCP-1 and IL-6 were also increased by treatment of cells with miR-134 mimic.^41^ In contrast, miR-467b could attenuate lipid accumulation and proinflammatory cytokine secretion by directly targeting LPL in RAW 264.7 macrophages.^42^

In the regulation of the cholesterol efflux of macrophages, miR-33a/b plays an important role in human and miR-33a in mice. miR-33a is highly conserved across species and encoded within an intron region of SREBP-2 in both humans and mice. However, miR-33b is embedded within the human SREBP-1. miR-33 negatively regulates cholesterol efflux by targeting ABCA1 at multiple miR-33 binding sites that are highly conserved across humans and large mammals.^43,44^ This phenomenon has been reported in mice, where the inhibition of miR-33a is combined with the increase of circulating HDLC levels.^45,46^ ABCG1 (in mice only) and Niemann-Pick C (NPC1, humans only) are also targets of miR-33 during cholesterol efflux regulation.^9^ In addition, it is noted that the cellular cholesterol level could significantly affect the expression of miR-33. The increase of the intracellular cholesterol content in macrophages and hepatic cells can cause a decrease of miR-33 and thus increase the cholesterol efflux.^47^ In mouse models, anti-miR-33 therapy has been shown to promote macrophages cholesterol efflux and ultimately reduce plaque progression or at least promote the regression of AS.^46,48,49^

Several miRNAs other than miR-33 also regulate the cholesterol metabolism in macrophages by targeting ABCA1 and/or ABCG1. As previously reported, miR-19b leads to a decrease of apoA^−/−^ mediated cholesterol efflux by suppressing the expression of ABCA1 in foam cells that differentiated from human THP-1 macrophages and mouse peritoneal macrophages.^8^ miR-19b also significantly affected macrophage RCT, plasma lipid profile, and atherosclerotic lesion area in apoE^−/−^ mice. miR-144 has been reported to inhibit the cholesterol efflux to apoA-I by directly targeting ABCA1 in mouse and human hepatocytes. Over-expression of miR-144 accelerates atherosclerosis and reduces RCT in vivo.^50,51^ miR-26, miR-27a, miR-758, and miR-148a have similar functions for repressing ABCA1 expression and cholesterol efflux.^52–55^ miR-10b also negatively regulates cholesterol efflux from murine- and human-derived macrophages by directly repressing the expression of ABCA1 and ABCG1.^56,57^ Wang et al. indicated that CoQ10 may increase macrophage RCT by regulating miR-378 to increase ABCG1 expression.^58^ However, no evidence exists that suggest a direct targeting of ABCG1 by miR-378.

### Dietary compounds targeting cholesterol metabolism-associated miRNAs of macrophage

Epidemiological studies have correlated the intake of diets rich in different compounds with the incidence of AS and cardiovascular disorders.^59,60^ Such as that unhealthy diet (rich in saturated fat, cholesterol, and salt as well as a low intake of fruit, vegetables, and fish) is one of the major underlying factors for AS. In contrast, a plant-based diet, which contains several chemical compounds such as polyphenols, plant sterols, and fibers can reduce the development and progression of AS.^61–63^ In recent years, the effects of some dietary compounds on AS-related macrophage have been illustrated at the level of miRNAs (Fig. 2).

Diosgenin (Dgn), as a naturally steroidal sapogenin, is present in a variety of plants, including fenugreek, yam root, and soy bean. It has cardiovascular protective activities and positively affects dyslipidemia.^64^ The lipid-lowering activity and the anti-atherogenic property of Dgn are related to the suppression of intestinal cholesterol absorption and the promotion of hepatic cholesterol secretion.^65^ Further study illustrated that Dgn enhanced ABCA1-dependent cholesterol efflux in human THP-1 macrophages by suppressing miR-19b. However, a detailed pathway for the regulation of miR-19b by Dgn has still not been reported.^66^

The coenzyme Q10 (ubiquinone, ubiquinol, or CoQ10) is a vitamin-like supplement that has profound inhibitory effects on the formation of foam cells differentiated from both mouse and human macrophage cells. CoQ10 upregulates ABCG1 expression by suppressing miR-378, and thus enhances cholesterol efflux from macrophages to HDL. The regulation of miR-378 by CoQ10 is mediated through c-Jun, a key heterodimeric partner of the AP-1 transcriptional complex. Therefore, the existence of an AP-1/miR-378/ABCG1 regulatory axis can be proposed that links CoQ10 to macrophage cholesterol efflux to HDL and atherosclerosis.^58,67^

Breakdown of anthocyanins to protocatechuic acid (PCA) by gut microbiota can accelerate the macrophage RCT in apoE^−/−^ mice via repressing miR-10b, which lead to the increase of ABCA1 and ABCG1 expression and finally, at least in part, result in the regression of established AS. The decrease of miR-10b caused by PCA may be caused by the inhibition of the CD44-activated Twist pathway via decreasing TNF-α production, partly verifying the cross-link between cholesterol metabolism and inflammation in AS.^57^

Betulinic acid (BA) is a naturally occurring triterpenoid that is widely distributed throughout the plant kingdom.^68^ It has been reported that BA can repress miR-33a/b and enhance ABCA1 expression. In this way, BA can promote the cholesterol efflux, reduce the levels of cellular cholesterol and cholesterol ester contents in macrophages and as a result, reducing AS lesion size.^69^ In addition, studies have shown that ABCA1 expression is reduced by exposure to inflammatory stimuli including IL-1β, TNF-α, and LPS in NF-κB dependent pathway.^70,71^ In this study, NF-κB was the first time to be reported that positively regulated the expression of miR-33s, and in apoE^−/−^ mice, BA inhibited NF-κB activation and then further down-regulated miR-33s expression.^69^

Epigallocatechin-3-gallate (EGCG) is one of the most popular polyphenols and is abundantly found in green tea, apple skins, plums, and onions.^72^ It can upregulate ABCA1 expression by decreasing miR-33a generation and thus reduce lipid accumulation of macrophage-derived foam cells as a result. This is one of the molecular mechanisms of the EGCG anti-atherosclerotic effect.^73^ In addition, EGCG could also inhibit atherosclerosis through affecting the Jagged-1/Notch pathway, which influenced by ox-LDL.^74^

In summary, these data provide evidence that the present natural dietary components play a key role in the regulation of different miRNA signaling pathways about the cholesterol efflux and thus could provide a novel therapeutic opportunity for the treatment of AS. However, it is noted that with regard to the cholesterol intake and intracellular metabolism of macrophage, the influence of functional dietary compounds on them via miRNAs still remains unexplored, which could be an important concern in the future study and a milestone for AS biology and therapeutic discovery.

## Dietary compounds inhibit AS by influencing the inflammatory response in macrophage via miRNA

Many reports have demonstrated that inflammatory mediators participate in all phases of AS, from lesion initiation through progression, and ultimately to clinical complications of this disease.^26^ In addition, the interaction between inflammation and cholesterol metabolism in AS makes it difficult to completely distinguish both pathways.

### Inflammatory response in macrophage

The presence of some inflammatory factors can increase the development of AS. For example, interferon-γ (IFN-γ) can enhance the uptake of acLDL (acetylated LDL)/ox-LDL in macrophages by increasing SR-A/SR-PSOX expression and consequently promote the formation of foam cells. IFN-γ can also reduce the cholesterol efflux through the inhibition of ABCA1, ABCG1, and apoE expression, and elevate the intracellular cholesteryl ester levels by increasing the ACAT1 expression.^75^ The tumor necrosis factor α (TNF-α) can repress ABCA1 and ABCG1 expression and enhance ACAT1 expression, thus promoting the development of AS by enhancing the trap of ox-LDL in foam cells and the accumulation of cholesteryl esters.^76^ TNF-α and interleukin 6 (IL-6) can promote AS by exciting the expression of SR-AI, which results in the increase of cholesterol uptake process in macrophages.^77^

However, some cytokines can inhibit the development of AS via an inhibition of inflammation and the trap of ox-LDL. For example, TGF-β1 inhibits ox-LDL uptake in THP-1 macrophages and the formation of foam cells by repressing CD36/SR-A expression.^78,79^ IL-10 inhibits murine AS in vivo via down-regulation of CD36 expression combined with enhancing ABCA-1/ABCG-1 expression.^80^ The nuclear factor κB (NF-κB) inhibits the efflux of cholesterol from macrophages to form HDL by reducing the expression of apoAI and ABCA1.^81^ Inhibition of NF-κB transcriptional activity can significantly reduce foam cell formation induced by TNF-α.^77^ In contrast, some AS risk factors such as inflammatory cytokines, ox-LDL, angiotensin II, and hemodynamic forces can activate NF-κB signaling. The activated NF-κB signaling leads to the increased expression of proinflammatory genes, including cytokines, adhesion molecules, and chemokines.^82^ The exposure to inflammatory stimuli including IL-1β, TNF-α, and LPS in the NF-κB dependent pathway can reduce ABCA1 expression.^70,71^

Furthermore, the cholesterol metabolism can also regulate the inflammatory response. Cholesterol itself is a type of inflammatory cytokine that can induce an inflammatory response by activating the p38 MAPK signal pathway via TLR3 and/or TLR4 signaling.^83^ Via combination with CD36 on the surface of the macrophage, ox-LDL can induce the activation of NF-κB, increasing the synthesis and secretion of IL-1β, and inhibiting the production of the anti-inflammatory molecule IL-10.^84^ As a key factor for RCT, HDL is also an anti-inflammatory molecule. It can relieve the expression of MCP-1, induced via cellular inflammatory signaling, to inhibit the chemotaxis of monocytes. As the principal component of RCT, apoAI can decrease the expression and activation of CD11b to relieve inflammation through MyD88-dependent TLR signaling.^85^ ABCA1 has also been reported to exhibit a protective effect on the inflammatory response induced by cytokines.^86^

Therefore, the factors that influence inflammation also tend to affect the cholesterol metabolism. More importantly, many food compounds exhibit their AS inhibition effects through affecting the inflammation signaling.

### miRNAs involved in macrophage-related inflammation

In addition to influencing the cholesterol metabolism, miRNAs can also regulate AS via inflammation signaling. Anti-inflammatory miRNAs have gained significant attention due to their therapeutic application in AS. Some miRNAs, such as miR-155 have even been implicated in the regulation of both of macrophage inflammation and the cholesterol metabolism, especially the uptake of ox-LDL.^11,19,38,87^

The miR-146 family (miR-146a/b) can regulate the IL-1 receptor associated kinase-1 (IRAK1), and the TNF-receptor associated factor-6 (TRAF6). IRAK1 and TRAF6 activate the downstream transcription factors NF-κB and AP-1 and thus upregulate the TLR4-mediated immune response.^88^ Recently, ApoE is identified to directly upregulate macrophage miR-146a, which in turn represses NF-κB signaling cascades via direct inhibition of miR-146a target genes IRAK1 and TRAF6. A further evidence shows that overexpression of miR-146 leads to the down-regulation of NF-κB activation and then reduces AS.^36^ It has been reported that miR-146 is a critical brake on pro-inflammatory NF-κB signaling in several cell types, including ECs and bone marrow-derived cells. Elevating miR-146a expression in the vasculature can inhibit endothelial activation and atherogenesis through restraining NF-κB signaling.^89^ Overall, upregulation of miR-146 can inhibit the development of AS.

miR-155 is the other one and well-known miRNA related to atherosclerotic inflammation. The role of miR-155 in AS varies during different stages of the atherosclerotic development. Inflammatory factors in macrophages, including ox-LDL, IFNγ, and the activation of TLRs, can induce the expression of miR-155, which in turn promotes the expression of inflammatory factors (such as IL-5, NOS2, and TNF-α) and thus enhances cellular inflammatory effects.^90^ miR-155 can regulate downstream signal pathways such as proinflammatory NF-κB signaling by targeting several mRNAs in macrophages, including SMAD2, FOXO3a, SOCS1, SHIP1, and BCL6.^11,19,87^ miR-155 also mediates the downstream phosphorylation of MAPK and STAT1 via targeted proteins such as SOCS1, PU1, and SHIP1, eventually reducing production of many cytokines, such as IL-2, IL-6, and IFN-γ. Among these proteins, SOCS1 is a negative feedback inhibitor for cytokine signaling. It disrupts inflammation by inhibiting the NF-κB signaling pathway.^91^ In ox-LDL-stimulated THP-1 macrophages, miR-155 was markedly upregulated in a dose-dependent manner. Targeting miR-155 promoted the NF-κB nuclear translocation and potentiated the NF-κB driven transcription activity.^38^ In summary, it can be concluded that miR-155 plays a key role in the atherogenic programming of macrophages, which both sustains and enhances inflammation and can thus be developed as a potential therapeutic target to treat AS.

In addition to the miRNAs mentioned above, miR-125a-5p was also found to partly regulate the inflammatory response in ox-LDL-treated monocyte/macrophages. The inhibitor of miR-125a-5p can significantly increase the secretion of IL-2, IL-6, TNF-a, and TGF-β in the THP-1 macrophage.^37^ In addition, miR-467b can not only regulate the macrophage cholesterol metabolism via targeting ACAT1 3′UTR,^39^ but can also decrease TNF-α and monocyte chemoattractant protein-1 (MCP-1) levels in RAW 264.7 macrophage and ultimately play an atheroprotective role in AS.^42^

### Dietary compounds inhibit AS via inflammation-associated miRNAs of macrophage

During earlier years, many dietary compounds have been found to inhibit the occurrence of inflammation phenomena. Currently, it has been suggested that many dietary compounds perform this function via regulating inflammatory miRNAs, especially those in AS related macrophages. Most of these functional dietary compounds belong to polyphenols. These widely reported compounds are listed as follows.

Quercetin is a flavonoid that can widely be found in human diets such as in onions, apples, tea, and red wine.^92,93^ It has been found to exhibit biological effects in the attenuation of atherosclerotic inflammation. Quercetin and its one major metabolite-isorhamnetin decrease the mRNA and protein levels of pro-inflammatory markers such as TNF-α, IL-1β, and IL-6, directly via down-regulation of miR-155 or by inhibiting the NF-κB pathway. These two compounds can also indirectly inhibit the NF-κB pathway by activating the Nrf2 signal transduction cascade. However, further studies are required to identify whether the Nrf2 signaling pathway may also be directly affected by miR-155, although it has been reported that Bach1, a repressor of Nrf2 signaling, is targeted by miR-155.^23^ Collectively, the finding that miR-155 can be regulated by dietary flavonoids provides a detailed insight into the mechanism of flavonoid-induced attenuation of inflammatory processes.

In addition to quercetin, allyl-isothiocyanate (AITC) was also reported to repress inflammation in RAW264.7 macrophages via attenuating miR-155. AITC is an aliphatic isothiocyanate that is derived from its precursor sinigrin and spreads widely throughout different brassica species including mustard, horseradish, and wasabi. It was found that AITC could attenuate the miR-155 levels in a dose-dependent manner and down-regulated some proinflammatory molecules including TNF-α, IL-1β, and iNOS. In addition, the anti-inflammatory effects of AITC were also found by an increase in Nrf2 nuclear translocation (negatively regulate NF-κB expression) and consequently increased the mRNA and protein levels of the Nrf2 target gene HO1, which finally down-regulated inflammation. Although the relationships between miR-155 and NF-κB and Nrf2 signaling pathway with AITC treatment were not discussed, the results about the aforementioned quercetin and isorhamnetin provide important information. It could be deduced, but has not been verified yet, that AITC performed its anti-inflammatory effect via miR-155, which targets the NF-κB and Nrf2 signaling pathway.^94^ Certainly, it has been noted that miR-27a, miR-142-5P, miR-153, miR-144,^95^ miR-93,^96^ and miR-28^97^ could also regulate the expression of Nrf2; however, whether these miRNAs also play an important role in macrophage-regulation by AITC is not known to date. In addition, some other food compounds such as sulforaphane,^98^ polyunsaturated fatty acids docosahexaenoic acid, and eicosapentaenoic acid^99^ could suppress LPS-induced inflammation in mouse peritoneal macrophage through Nrf2 dependent pathway. Whether this could be realized through the regulation these aforementioned miRNA also remains unknown. Therefore, from the view of miRNA-regulation to reveal the effect mechanism of food compounds on AS-related macrophage should be a promising breakthrough in the related research area.

Apigenin (4’,5,7-dihydroxyflavone) is an abundant flavonoid in many foods including parsley, celery, and chamomile tea.^100,101^ It has been certified to have anti-proliferative and anti-inflammatory activities.^102,103^ Intake of apigenin results in lower incidence of ovarian cancer and cardiovascular disorders.^59,104^ A recent study shows that apigenin can reduce LPS-induced miR-155 expression through inhibition of NF-κB in macrophages.^19^ Further research shows that as two targets of miR-155, FOXO3a (Forkhead Box O3a, an inhibitor of NF-κB),^105^ and SMAD2 (smooth-muscle-actin and MAD-related proteins 2, a suppressor of inflammatory molecules TNF-α and iNOS)^106^ are increased by apigenin targeted on the miR-155, resulting in the decrease of TNF-α and finally the inflammatory processes are attenuated.^19^

As another regulator of miR-155 in macrophages, curcumin was lastly found to play its anti-inflammatory role by inhibiting miR-155 through the phosphoinositide 3-kinase (PI3K)-AKT signaling pathway.^107^ Curcumin is a natural polyphenol, commonly used as a spice and food-coloring material. Numerous articles have reported that curcumin can promote the degradation of TNF-α and IL-6 expressed^108^ and the PI3K/AKT signaling pathway plays a crucial role in the anti-inflammatory effects of curcumin in LPS-activated inflammatory responses. In a recent study, the phosphorylation of PI3K, p85a, and AKT levels decreased significantly in curcumin treatment group and the PI3K/AKT pathway is verified to be required for the inhibition of miR-155.^109^

Many other dietary compounds also perform their anti-inflammatory effects via miR-155. For example, resveratrol, a polyphenol rich in grapes, alleviates LPS-elicited inflammation in RAW264.7 macrophages by suppressing miR-155 and concurrently boosting SOCS1 production.^110^ Genistein, the major active isoflavonoid in soybeans, reverses ox-LDL-induced inflammation via miR-155/SOCS1-mediated repression of the NF-κB signaling in human umbilical vein ECs (HUVECs).^111^

In addition to miR-155, other miRNAs are also important for the regulation of macrophage inflammation by dietary compounds.^36^ For example, in human THP-1 macrophage as well as male mice, coenzyme Q10 fine-tunes the inflammatory response via moderating the reduction of miR-146a by targeting TRAF6, which is a regulator protein within the TLR-signaling pathway for the formation and accumulation of reactive oxygen species that tends to promote the development of AS.^112^

Overall, the currently reported miRNAs that are related to the inhibition of AS by dietary compounds through inflammation signaling are mainly miR-155 and miR-146a, much less than that related to cholesterol metabolism in AS. Furthermore, the miRNAs correlated to the inflammatory responses in macrophage foam cells have not been considered in studies on food compounds, such as miR-33s, miR-125a-5p, and miR-467b.

## Conclusion

Until now, numerous miRNAs have been identified to be related to the regulation of AS in aspects of cholesterol metabolism (15 miRNAs) and inflammation signaling (4 miRNAs) of macrophages. Among these, only five miRNAs in the cholesterol metabolism (miR-19b, miR-378, miR-10b, miR-33a, and miR-33b) and two miRNAs related to inflammation signaling (miR-155 and miR-146a) have been reported to be regulated by food compounds. In the cholesterol metabolism, a strong diversion was found in the type of miRNAs among different food compounds, such as diosgenin targets miR-19b, while coenzyme Q10 conducts its function through miR-378; betulinic acid influences miR-33a/b, and epigallocatecin-3-gallate inhibits AS via miR-33a; PCA performs the cholesterol metabolism influencing activity by targeting miR-10b. However, for inflammation signaling, most food compounds target miR-155, such as quercetin, ally-isothiocyanate, apigenin, curcumin, and genisin. Only coenzyme Q10 performs its inflammation inhibition effect via miR-146a. It can also be seen that the same food compound (such as coenzyme Q10) performs the AS inhibition effect via both cholesterol metabolism and inflammation signaling pathways by targeting miR-378 and miR-146a, respectively. Overall, food compounds perform their inhibitory effects on AS via different pathways and different miRNAs.

Furthermore, it should be mentioned that many receptors are involved in the effects of food compounds on AS via miRNAs. Among these, ABCA1 and ABCG1 are the major receptors that are involved in the cholesterol metabolism, while NF-κB and Nrf2 signaling and PI3K/AKT signaling are the most frequently involved pathways in inflammation related to the inhibition of food compounds on AS.

The currently available studies provide important guidance for the therapeutic treatment of AS and even CVD at the molecular level. However, further studies are still urgently required in this field, since many AS related miRNAs have not been studied with respect to food compounds. Potential projects that require further study include: (1) The influence of food compounds on macrophage cholesterol intake via miRNAs; this may be a milestone in AS biology and therapeutic discovery, because excessive cholesterol influx is the principal reason for foam cell formation. (2) The molecular mechanism of food compounds modulating miRNAs remains unknown. Currently, there is no direct evidence to illustrate the mechanism of polyphenol regulation of the miRNA, although some polyphenols have been found to bind to mRNAs and proteins. Additionally, some miRNAs are intronic of genes and polyphenols may affect the miRNA levels via modifying expression of their host gene. (3) The relationships between the structure of food compounds and their regulation of miRNA need to be identified. A great diversity exists among the structure of different food compounds. Only a small part of these, and mainly polyphenol and flavonoids, have been tested so far. It is necessary to understand the relationship between the structure and its activity to modulate miRNAs, which is of great significance to the exploitation of these natural resources. In summary, more studies are required to find more effective, natural, and safe compounds for AS prevention and therapy.

### Data availability

Data sharing not applicable to this article as no datasets were generated or analyzed during the current study.
